# Neural dynamics underlying coherent motion perception in children and adults

**DOI:** 10.1016/j.dcn.2019.100670

**Published:** 2019-06-13

**Authors:** Catherine Manning, Blair Kaneshiro, Peter J. Kohler, Mihaela Duta, Gaia Scerif, Anthony M. Norcia

**Affiliations:** a Department of Experimental Psychology, University of Oxford, Anna Watts Building, Radcliffe Observatory Quarter, Woodstock Road, Oxford, OX2 6GG, UK; b Department of Otolaryngology Head and Neck Surgery, Stanford University School of Medicine, Stanford University, 2452 Watson Court, Palo Alto, CA, 94303, USA; c Department of Psychology, Stanford University, Jordan Hall, 450 Serra Mall, Stanford, CA, 94305, USA

**Keywords:** Evoked potentials, Visual development, Motion perception, Direction perception, Electroencephalography, Component decomposition

## Abstract

Motion sensitivity increases during childhood, but little is known about the neural correlates. Most studies investigating children’s evoked responses have not dissociated direction-specific and non-direction-specific responses. To isolate direction-specific responses, we presented coherently moving dot stimuli preceded by incoherent motion, to 6- to 7-year-olds (*n* = 34), 8- to 10-year-olds (*n* = 34), 10- to 12-year-olds (*n* = 34) and adults (*n* = 20). Participants reported the coherent motion direction while high-density EEG was recorded. Using a data-driven approach, we identified two stimulus-locked EEG components with distinct topographies: an early component with an occipital topography likely reflecting sensory encoding and a later, sustained positive component over centro-parietal electrodes that we attribute to decision-related processes. The component waveforms showed clear age-related differences. In the early, occipital component, all groups showed a negativity peaking at ˜300 ms, like the previously reported coherent-motion N2. However, the children, unlike adults, showed an additional positive peak at ˜200 ms, suggesting differential stimulus encoding. The later positivity in the centro-parietal component rose more steeply for adults than for the youngest children, likely reflecting age-related speeding of decision-making. We conclude that children’s protracted development of coherent motion sensitivity is associated with maturation of both early sensory and later decision-related processes.

## Highlights

• We measured evoked responses to directional motion in children and adults.
• Two reliable components with distinct time-courses and topographies were identified.
• Both component waveforms exhibited clear age-related differences.
• Both early sensory and later decisional processes seem to develop across childhood.

## 1 Introduction

Perceptual sensitivity to motion increases during development, with many aspects of motion processing continuing to develop through childhood (e.g., speed discrimination: Manning et al., 2012; coherent motion perception: Gunn et al., 2002; Manning et al., 2014; biological motion discrimination: Hadad et al., 2011). It has been suggested that motion sensitivity develops more gradually than sensitivity to form, and that this slow development may render it particularly vulnerable to atypical development in a range of neurodevelopmental disorders (Braddick et al., 2003). Yet, relatively little is known about the neural responses underlying the *typical* development of motion sensitivity in children: a crucial link required to understand how atypical neural development contributes to altered motion sensitivity in different disorders.

Evoked potentials are useful for studying the development of the neural correlates of motion processing, as they can be obtained relatively easily across the lifespan, from infancy to adulthood, and provide rich information about the time-course of motion processing. The research characterising motion evoked potentials in adulthood is extensive. In adults, stimuli that begin to move (typically after a period of stationary presentation) give rise to three distinct peaks (see Kuba et al., 2007, and Heinrich, 2007, for review). The first of these is a positive peak at around 130 ms after motion onset (P1), and the second is a negative peak at around 150–200 ms (N2). The P1 and N2 are observed in occipital electrodes, including Oz and electrodes positioned laterally on either side. A second positive peak can be found at the vertex with a latency of about 240 ms (P2), particularly for complex stimuli such as expanding or contracting optic flow. The relative dominance of these peaks varies with stimulus and task parameters, and there is more individual variability in the N2 than in the earlier P1 (Kuba et al., 2007).

The N2 is considered to be largely ‘motion-specific’, unlike the earlier ‘pattern-specific’ P1. The key distinction here is that motion-specific mechanisms capture information about motion direction rather than merely reflecting dynamic changes in luminance (Clifford and Ibbotson, 2003; Heinrich, 2007). The N2 is relatively contrast-independent (Bach and Ullrich, 1997), shows direction-specific adaptation (Hoffmann et al., 2001), and is thought to arise from extrastriate temporo-occipital areas, such as V5/MT and V3 (Aspell et al., 2005; Probst et al., 1993). In an attempt to isolate direction-selective mechanisms, some researchers have measured evoked activity in response to the onset of coherent, directional motion after a period of incoherent motion (Niedeggen and Wist, 1998, 1999; Patzwahl and Zanker, 2000). Here, the P1 component is no longer apparent in adults, but N2 can be measured from electrode Oz and neighbouring electrodes on either side, beginning at 130 ms after coherence onset and peaking at about 300 ms, followed by a slow positive shift that may be associated with the perceptual decision (Niedeggen and Wist, 1999). The amplitude of the N2 scales with the coherence of motion, with low levels of coherence failing to elicit a reliable evoked potential (Niedeggen and Wist, 1999; Patzwahl and Zanker, 2000).

But how do these distinct motion-evoked responses develop? When studying infants, it is difficult to obtain the large number of trials typically used for averaging when measuring adult evoked responses. The steady-state visual evoked potential technique offers increased signal-to-noise ratio (Norcia et al., 2015), allowing researchers to sidestep this problem and uncover direction-specific evoked responses which emerge as early as 6–10 weeks of age (Braddick et al., 2005; Wattam-Bell, 1991; Birch et al., 2000) and develop through infancy (Gilmore et al., 2007; Hou et al., 2009; Lee et al., 2013; Wattam-Bell, 1991). These studies have isolated direction-specific responses by focusing on responses to periodic alternations between motion directions (e.g., Gilmore et al., 2007; Wattam-Bell, 1991) or between coherent and incoherent motion (e.g., Hou et al., 2009).

However, there is relatively little research charting the development of direction-specific evoked responses through childhood. Children show immaturities in their responses to the onset of motion following a stationary stimulus, with the dominance of the P1 component and the latency of the N2 component reducing between 6 years and adulthood (Langrová et al., 2006). Moreover, these motion-onset potentials appear to develop more gradually than responses evoked by colour changes (Coch et al., 2005; Mitchell and Neville, 2004), and show considerable variability between children (Kubová et al., 2014). However, these studies have not isolated direction-specific responses, as, with the transition from stationary to moving stimuli, the onset of motion coincided with the onset of spatiotemporal luminance modulation. Gilmore et al. (2016) addressed this limitation and found differences in steady-state visual evoked responses to coherence-modulating optic flow motion in 4- to 8-year-olds (*n* = 33) compared to adults. While these results suggest that motion-specific evoked responses are still developing through early childhood, behavioural improvements in coherent motion processing continue past 8 years of age (Hadad et al., 2011; Manning et al., 2014). We would therefore expect the neural correlates of motion processing to similarly continue to develop into later childhood.

The goal of the current study was to use high-density EEG to characterise age-related differences in direction-specific evoked responses in a large sample of 6- to 12-year-old children and adults. We isolated direction-specific responses using the same approach as previous studies (Niedeggen and Wist, 1998, 1999; Patzwahl and Zanker, 2000), whereby participants were presented with an initial ‘boil’ period of incoherent motion, followed by coherent motion. Additionally, to extend and complement studies that have focused on averaged waveforms in certain electrodes, here we used the entire sensor array to identify maximally reliable components with a data-driven component decomposition technique that has been used successfully to investigate motion perception in adults (Dmochowski and Norcia, 2015). We predicted that evoked responses to the onset of coherent motion would develop gradually between 6 and 12 years, coinciding with behavioural improvements in accuracy and response time.

## 2 Methods

### 2.1 Participants

Participants were 102 children aged between 6 and 12 years and 20 adults aged between 18 and 35 years (9 females), with no reported history of developmental conditions and normal or corrected-to-normal vision (assessed with a Snellen chart). Children were recruited primarily through local schools and invitations to previously participating families, and adult participants were recruited through the University’s research participation scheme. The children were divided into three equally-sized age groups (*n* = 34): 6- to 7-year-olds (*M* = 6.79 years, *SD* = .56; 15 females), 8- to 10-year-olds (*M* = 9.20 years, *SD* = .68; 17 females) and 10- to 12-year-olds (*M* = 11.52 years, *SD* = .79; 15 females).

### 2.2 Apparatus

The experimental task was presented on a Dell Precision M3800 laptop (2048 × 152 pixels, 60 Hz) using MATLAB (Mathworks, MA, USA) and the Psychophysics Toolbox (Brainard, 1997; Kleiner et al., 2007; Pelli, 1997). EEG signals were acquired with a 128-electrode Hydrocel Geodesic Sensor Net connected to Net Amps 300 (Electrical Geodesics Inc., OR, USA), using NetStation 4.5 software. A photodiode was attached to the monitor to independently verify the timing of stimulus presentation. Participants made their responses using a Cedrus RB-540 response box (Cedrus, CA, USA).

### 2.3 Stimuli

**Fig. 1:**
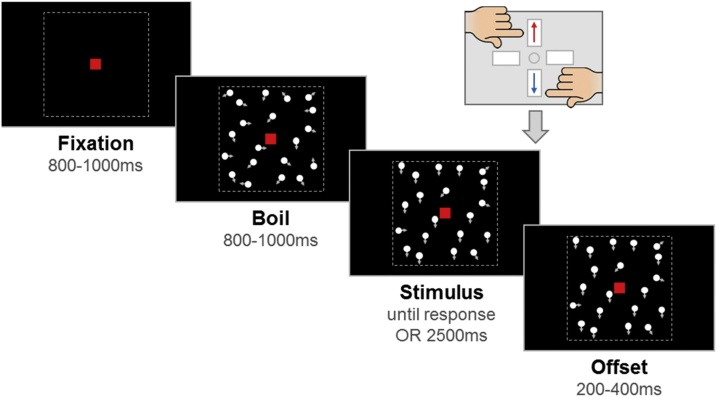
**Schematic representation of trial procedure.** An initial *fixation* period was followed by a *boil* period consisting of random, incoherent motion, which was in turn followed by a *stimulus* period. The stimulus period contained upward or downward coherent motion and the participant was required to report the direction using a response box. If there was no response, the stimulus remained on the screen for 2500 ms. The stimulus remained on the screen for an *offset* period after the response or after the maximum stimulus duration was reached. Note that arrows (indicating movement) and dotted lines (marking the square stimulus region) are presented for illustrative purposes and were not visible in the task.

One hundred white stimulus dots (diameter 0.19°; luminance 248 cd/m^2^) were randomly positioned within a central square region (10° × 10°) on a black screen (luminance 0.22 cd/m^2^) and moved at a speed of 6˚/s. The dots had a limited lifetime of 200 ms (with randomised starting lifetimes), and dots moving outside the square stimulus region were wrapped around to the opposite side. A central red fixation square (0.24° × 0.24°) was present on the screen throughout the trial. Each trial consisted of a fixation period, a boil period, a stimulus period, and an offset period (see Fig. 1 and stimulus videos). The fixation period, during which only the central fixation square was shown, was presented for a randomly selected duration between 800 and 1000 ms. The stimulus dots first appeared in the boil period, during which they moved in random, incoherent directions, for a randomly selected duration between 800 and 1000 ms. In the stimulus period, a proportion of the dots moved coherently either upward or downward, while the remainder of the dots continued to move in random directions. The stimulus period lasted until a response was made, or until 2500 ms had passed. Finally, an offset period continued the coherent stimulus presentation for a randomly selected duration between 200–400 ms. The jittered durations of the fixation, boil and offset periods were intended to minimise expectancy effects.

### 2.4 Experimental task procedure

Participants played a game with 10 ‘levels’ (each corresponding to a trial block) in which they were asked to report the direction of ‘fireflies’ (white stimulus dots) using one of two keys (up, down) on a response box, as quickly and accurately as possible, in line with previous studies of perceptual decision-making (e.g, Dmochowski and Norcia, 2015; Kelly and O’Connell, 2013; O’Connell et al., 2012; Twomey et al., 2015). Block 1 was a training phase including 4 demonstration trials, up to 20 criterion trials, and 8 practice trials. The demonstration trials were used to explain the task to participants. They had no boil phase and an unlimited stimulus phase, and became more difficult (100%, 100%, 75% and 50% coherence). The criterion and practice trials included a boil phase and a maximum stimulus phase duration of 2500 ms. In the criterion trials, the dots moved with 95% coherence in the stimulus phase, and participants were required to meet a criterion of 4 consecutive correct responses within 20 trials in order to continue with the task. The practice trials increased in difficulty from 80% to 10% stimulus coherence in 10% steps. Participants were reassured if they got some of these wrong and/or if they had to guess. Throughout the training phase, trial-by-trial feedback was presented (“That was correct!”, “It was the other way that time”, “Timeout! Try to be quicker next time!”).

The experimental phase consisted of 324 trials across blocks 2 to 10. These trials included 36 repetitions of each of four coherence levels (10%, 30%, 50%, 75%), for each coherent direction (upward, downwards). We also included 18 catch trials with 100% coherent motion for each coherent direction. No trial-by-trial feedback was given in the experimental phase, apart from a ‘timeout’ warning if no response was provided within 2500 ms. Participants took a short break at the end of each block and were given points for the preceding block of trials. The points reflected both accuracy and response time (1 / median response time * accuracy, rounded to the nearest integer). Trials were presented automatically in block randomised order, although the experimenter had controls to pause and resume trial presentation. Two 6-year-old participants did not complete all of the blocks in the experimental phase, instead completing 180 and 216 trials, respectively.

### 2.5 General procedure

The procedure was approved by the local research ethics committee board. Adult participants and parents of child participants provided written informed consent, and children provided verbal or written assent. Participants first completed a Snellen acuity test to confirm normal or corrected-to-normal vision. The sensor net was then placed on the head, and adjustments were made to ensure electrode impedances were below 50 kΩ. EEG data were acquired at a sampling rate of 500 Hz with a vertex reference electrode. Participants then sat 80 cm away from the computer screen in a dark room for the experimental task. Children were closely monitored by a researcher sitting beside them. The researcher provided general encouragement and task reminders, pausing before the start of a trial where necessary (e.g., to remind the child to keep still). Children had short breaks at the end of each block, and a longer break halfway through (i.e., at the end of block 5), at which point the EEG recording was paused, electrode impedances were re-assessed and adjustments were made if required to bring electrode impedances below 50 kΩ. Note that for two children, impedance checks were completed instead at the end of block 4 or block 6. Children marked their progress through the blocks using a stamper on a record card. The whole session took no longer than 1.5 h. Adult participants were paid £15 and children were given a £5 gift voucher to thank them for their time. The experimental code and analysis code can be found at https://osf.io/fkjt6/.

### 2.6 EEG data pre-processing

EEG signals were band-pass filtered offline between 0.3 and 40 Hz using NetStation’s filters, before being exported as a binary file for further pre-processing using MATLAB. We applied an 8th-order, zero-phase Butterworth highpass (0.3 Hz) filter as low frequencies were not being sufficiently attenuated by the NetStation filter. The data were epoched into trials, defined from the onset of the fixation period to the end of the offset period, and the data from each trial were median-corrected for DC offsets. Next we identified bad electrodes across each half of the session (before and after the break when impedances were re-checked). We plotted a histogram of the absolute amplitude of each participant’s data across samples and electrodes and identified outliers as those exceeding the 97.5th percentile. We removed electrodes from a half-session if they contained 15% or more samples exceeding the 97.5th percentile and replaced them with an average of the nearest neighbouring electrodes (*M* = 1.69% electrodes replaced per participant; range = 0–5.08%). For most electrodes, 6 neighbours were used, whereas electrodes on the perimeter of the net were replaced with the average of the 4 nearest neighbours. We then regressed out horizontal and vertical electrooculogram (EOG) from each electrode. The horizontal EOG was calculated as the difference between the electrodes in the right and left outer canthi (electrodes 125 and 128) and the vertical EOG was determined as the difference between the sum of electrodes positioned above the eyes (8, 25) and the sum of those placed on the cheek (126, 127). We then replotted amplitude histograms for each individual and recalculated the 97.5th percentile, removing electrodes for each trial in which 15% of samples or more exceeded this cut-off (*M* = 3.63% data removed per participant, range = 0.02–6.16%). Next we removed transients – defined as samples that were four or more standard deviations away from the electrode’s mean – and replaced these with missing values. The data were converted to the average reference and baselined to the average of the last 100 ms of the boil period. In trials where data were removed from 19 or more (≥ 15%) electrodes, we excluded the EEG data completely (*M* = 5.93% trials removed per participant, range = 0–14.81%). We also removed the data from two electrodes which showed unusually flat activity during the whole recording for a participant in the 6- to 7-year-old group.

### 2.7 EEG analysis

A dimensionality reduction technique – reliable components analysis (RCA; Dmochowski et al., 2012, 2014; Dmochowski and Norcia, 2015) – was used to identify components that maximised spatiotemporal trial-to-trial reliability. Similar to principal components analysis (PCA), this method computes sets of electrode weights for each component. However, whereas PCA components maximise variance explained, RCA components maximise trial-to-trial covariance of the EEG, and can lead to an increased signal-to-noise ratio (Dmochowski et al., 2015). The trial-to-trial covariance criterion is appropriate for studying evoked responses as components of interest are expected to be spatiotemporally reproducible across trials – in line with the motivation of standard event-related potential research which computes grand averages across trials. A forward-model projection of the weights can be used to visualise components as scalp topographies (Haufe et al., 2014; Parra et al., 2005), and data projected through these weights can be averaged for each timepoint to provide a time course for the component which can be compared across groups and conditions. This method has advantages over traditional event-related potential analysis as it uses a data-driven approach to identify topographic regions of interest using the whole electrode array while increasing the signal-to-noise ratio as each component represents a weighted average of electrodes. This technique has previously been used to investigate evoked responses to fine motion stimuli in adults (Dmochowski and Norcia, 2015) and to compare steady-state motion responses in infants and adults (Kohler et al., 2018).

We selected trial data from 100 ms prior to the stimulus onset to 600 ms following the stimulus onset. We initially applied RCA to these stimulus-locked data for each group separately. The first two stimulus-locked components extracted by RCA in the adult participants together explained 76% of the total trial-by-trial reliability in these participants (61% for component 1 and an additional 15% for component 2). We therefore focused on these components for our analysis. The topography of component 1 was similar for children and adults, while components 2 and 3 exhibited some differences across the age groups (see Supplementary Fig. S1). To conduct age-related comparisons, we projected the child data through the component 1 and component 2 weights derived for the adults and averaged these to provide component waveforms. This approach allowed us to directly compare the response dynamics for each component across the age groups, and characterise how ‘adult-like’ the responses in the child groups were. To more extensively characterise the temporal evolution of the components, we projected a longer record of stimulus-locked data through the weights, spanning 100 ms preceding the stimulus onset to 800 ms after the stimulus onset. For some fast responses, the trial had already ended before the end of this time window, in which case these samples were left missing so that only data recorded during the coherent motion or stimulus offset period were included in the analysis.

We assessed the effects of group and coherence on the reliable component waveforms with a mass univariate approach, using the second-level analysis functions from the LIMO EEG toolbox (Pernet et al., 2011). This approach allowed us to assess effects at each timepoint, while using a temporal clustering technique to control for multiple comparisons (see Maris, 2012, and Pernet et al., 2015, for review). First, we centered the data for each group and coherence condition separately. Then, for each of 2000 bootstrap iterations, we randomly sampled with replacement the participants’ centered data and conducted a two-way ANOVA with group as a between-participants factor and coherence condition as a repeated measures factor, in order to get a distribution of *F* values expected under the null hypothesis (i.e., 2000 *F* values for each factor/interaction at each timepoint; Pernet et al., 2015). We then used cluster statistics to control the family-wise error rate (Maris and Oostenveld, 2007; Pernet et al., 2015). We clustered the significant (*p* <  .05) bootstrapped *F* values for each factor/interaction and used the maximum sum across clusters to derive a temporal cluster threshold for each factor/interaction with an alpha level of 0.05. Finally, we computed sums of temporal clusters of significant *F* values in the original, non-bootstrapped data and identified clusters that exceeded the cluster threshold. To test for group differences that vary with the level of coherence, we conducted one-way between-participant ANOVAs for each coherence condition using the same bootstrapping and clustering approach. We then further investigated these group effects using bootstrapped *t*-tests with clustering to test for differences between each of the child groups and the adult group.

To compare the results from our reliable components analysis with previous research, we also conducted a more traditional coherence-onset visual evoked potential analysis restricted to occipital electrodes. Niedeggen and Wist (1999) selected electrode Oz and three electrodes positioned 3 cm laterally to the right and to the left. In order to provide comparable results with our EEG system, we selected the activity in electrode 75 (corresponding to Oz) and the four laterally-positioned electrodes on either side (50, 58, 65, 70, 83, 90, 96, 101; see Fig. 5 for electrode positions).

## 3 Results

### 3.1 Age-related differences in behaviour: accuracy and response time

**Fig. 2:**
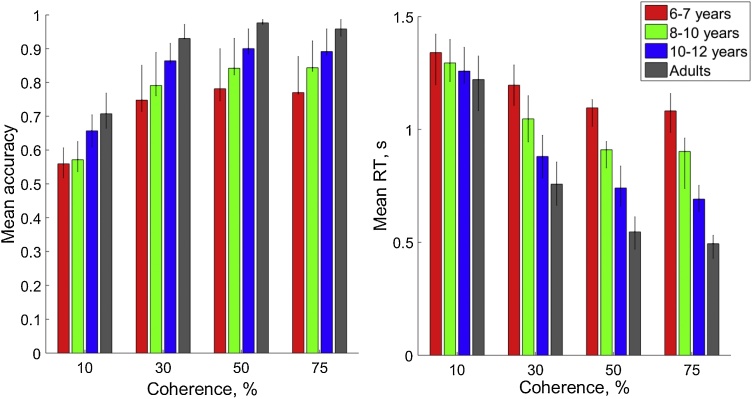
Mean response time and accuracy for each group. Error bars represent 95% confidence intervals.

As expected, there were effects of age-group and coherence condition, on both accuracy and response time (Fig. 2). There were age-related increases in accuracy, *F*(3, 118) = 11.71, *p* <  .001, η_p_^2^ = .23, with planned contrasts showing that 6- to 7-year-olds (*M* = .72) and 8- to 10-year-olds (*M* = .76) made significantly less accurate responses than adults (*M* = .89; *p*s < .001), whereas the older, 10- to 12-year-old children (*M* = .83) differed only marginally significantly from the adults (*p* =  .051). The coherence condition also affected accuracy, *F*(2.04, 240.26)^2^  = 326.68, *p* <  .001, η_p_^2^ = .74, with less accurate responses in the 10% (*M* = .62) and 30% (*M* = .83) conditions than in the 75% coherence condition (*M* = .87; *p* < .001), but no significant difference between the 50% (*M* = .88) and 75% coherence condition (*p* = .145). The interaction between coherence and age-group was not significant, *F*(6.11, 240.26) = .94, *p* = .47, η_p_^2^ = .02.

Mean response time also showed significant effects of group, *F*(3, 118) = 25.50, *p* < .001, η_p_^2^ = .39, and coherence, *F*(1.39, 164.00) = 521.58, *p* < .001, η_p_^2^ = .82. All groups of children made significantly slower responses than the adults (6- to 7-years: *M* = 1.18 s, *p* <  .001; 8- to 10-years: *M =* 1.04 s, *p* <  .001; 10- to 12-years: *M* = .89 s, *p* = .011; adults: *M* = 0.75 s). The highest coherence condition (*M* = .79 s) produced faster responses than the lower coherence conditions (10%: *M* = 1.28 s, *p* < .001; 30%: *M* = .97 s, *p* <  .001; 50%: *M* = .82 s, *p* <  .001). Additionally, there was a significant interaction between coherence and group, *F*(4.17, 164.00) = 20.79, *p* <  .001, η_p_^2^ = .35. Follow-up one-way ANOVAs for each coherence condition showed that the effect of group on response time was not significant at the lowest (10%) coherence level, *F*(3, 118) = 1.43, *p* =  .24, η_p_^2^ = .04, but became more pronounced at higher coherence levels (30%: *F*(3, 118) = 27.19, *p* <  .001, η_p_^2^ = .41; 50%: *F*(3, 118) = 37.11, *p* <  .001, η_p_^2^ = .49; 75%: *F*(3, 118) = 36.37, *p* <  .001, η_p_^2^ = .48).

### 3.2 Age-related differences in EEG activity: reliable components

**Fig. 3:**
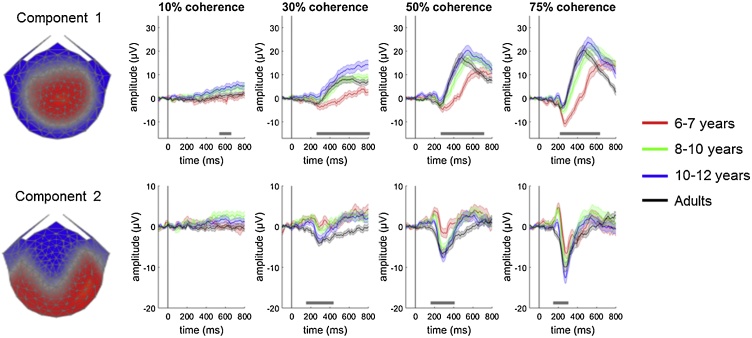
**Scalp topographies and waveforms for components 1 (upper panel) and 2 (lower panel).** Topographic visualisations of the forward-model projections of components 1 and 2 (left) reflecting the weights given to each electrode following reliable components analysis (RCA) on the adult data, pooled across coherence conditions. The waveforms (right) show the data multiplied by these spatial weights, for each group (red: 6- to 7-year-olds; green: 8- to 10-year-olds; blue: 10- to 12-year-olds; black: adults) and coherence condition. Shaded error bars represent the standard error of the mean. The grey horizontal bars show significant cluster-level effects of group for each coherence condition.

Topographic visualisations of the forward-model projections of the two most reliable components computed from the adult data using RCA are shown in Fig. 3, along with the averaged component-space EEG data for each age group at each coherence level. The component explaining the largest portion of the reliability (component 1) exhibited spatial maxima over centro-parietal electrodes and was characterised by a gradual increase in positivity beginning ˜250 ms after coherent motion onset (Fig. 3; upper panel). First we used a bootstrapped ANOVA with cluster-correction to investigate the effects of group and coherence on the temporal dynamics of this component. There was a significant cluster for the between-participants effect of group between 244 ms and 800 ms, and significant clusters for the within-participants effect of coherence between 206 ms and 298 ms and between 336 ms and 800 ms. The youngest children had lower component amplitudes and the component appeared to reach a peak later than in the older children and adults (see Fig. 3). Moreover, higher coherence levels were associated with higher component amplitudes within these time windows. There were also two significant clusters for an interaction between group and coherence between 244 ms and 548 ms and between 616 ms and 800 ms.

To assess the effects of group for each coherence condition separately, we conducted one-way bootstrapped, cluster-corrected ANOVAs. These analyses revealed that there were significant cluster-level effects of group for all coherence conditions (Table 1). The significant clusters for the effect of group are shown in Fig. 3. Within these clusters, we tested for cluster-level differences between the adults and each of the child age groups, to get an idea of at which age performance is adult-like (Table 1). The youngest, 6- to 7-year-old children had lower amplitudes than adults across a relatively broad time window, for the 30%, 50% and 75% coherence conditions. The oldest, 10- to 12-year-old children had higher amplitudes than the adults towards the end of the trial (after 600 ms) in the 10%, 30% and 50% coherence conditions. In contrast, the 8- to 10-year-olds showed no cluster-level differences compared to the adults in these conditions and differed from adults only in the 75% coherence condition between 284 and 428 ms, with lower amplitudes. It appears then that the amplitudes increase throughout childhood and then decrease again slightly in the adult group.

**Table 1:** Significant clusters for group comparisons in reliable components for each coherence condition.

	Component 1: Significant clusters (ms)				Component 2: Significant clusters (ms)			
Coherence:	10%	30%	50%	75%	10%	30%	50%	75%
*F*	552 – 646	276 – 800	280 – 702	232 – 620	None	166 – 422	174 – 394	162 – 290
*t*								
6-7 years vs. adults	None	322 – 700	300 – 572	234 – 550		166 – 422	174 – 394	162 – 284
8-10 years vs. adults	None	None	None	284 – 428		166 – 422	174 – 250	162 – 238
10-12 years vs. adults	602 – 646	640 – 800	608 – 702	None		166 – 236	None	166 – 206

Note. No significant clusters for the effect of group were found in the 10% coherence condition for component 2, so we did not conduct pairwise comparisons between groups for this coherence level.

Inspection of Fig. 3 also suggested that the slope of increasing positivity became steeper as a function of age. To quantify this, we fit a linear regression to each observer’s average component waveform between 260 ms and 460 ms after stimulus onset in each coherence condition and conducted a mixed group by coherence condition ANOVA on the slope coefficients. This revealed a main effect of coherence, *F*(2.08, 245.93) = 8.90, *p* < .001, η_p_^2^ = .07, with shallower slopes in the 10% coherence condition (*M* = 1.30, *SE* = .81) than in the higher coherence conditions (30%: *M* = 4.68, *SE* = .60, *p* <  .001; 50%: *M* = 4.51, *SE* = .32, *p* <  .001; 75%: *M* = 3.83, *SE* = .25, *p* =  .004). There was also a significant main effect of group, *F*(3, 118) = 4.22, *p* =  .007, η_p_^2^ = .10, with the youngest, 6- to 7-year-old children having shallower slopes (*M* = 2.03, *SE* = .54) than the adults (*M* = 4.23, *SE* = .70), *p* =  .014, while the slopes of the older children did not differ significantly from those of adults (8- to 10-year-olds: *M* = 3.45, *SE* = 0.54, *p* =  .38; 10- to 12-year-olds: *M* = 4.60, *SE* = .54, *p* = .68). The interaction between group and coherence level was non-significant, *F*(6.25, 245.93) = .90, *p* =  .50, η_p_^2^ = .02.

The component explaining the second largest portion of reliability (component 2) exhibited maxima over right occipital electrodes (Fig. 3, lower panel). Although the waveforms in the lowest (10%) coherence condition were mainly flat, a positive peak (in the child groups) and a later, negative peak (in all groups) were clearly visible in the highest coherence conditions (50% and 75%). The dominance of the positive peak relative to the negative peak appeared to reduce with age. Cluster-based statistics revealed a significant cluster for the between-participants effect of group between 160 ms and 374 ms. There was a significant cluster for the main effect of coherence between 220 ms and 416 ms, with larger absolute component amplitudes associated with higher coherence. There were no significant clusters for the coherence by group interaction. One-way bootstrapped ANOVAs with cluster-correction showed no significant clusters for the effect of group at the lowest (10%) coherence level. The higher coherence levels all showed significant clusters reflecting group differences (Table 1; Fig. 3). As seen in Fig. 3, the clusters for group differences onset earlier in this component and were generally of shorter duration than those in component 1 (Fig. 3). Pairwise group comparisons yielded significant clusters when comparing adults with the 6- to 7-year-old children and 8- to 10-year-old children for the 30%, 50% and 75% coherence conditions (Table 1). Even the oldest, 10- to 12-year-old children showed apparent immaturities in this component, showing significant differences from the adults in the 30% and 75% coherence conditions.

### 3.3 Comparison to the coherence onset visual evoked potential

**Fig. 4:**
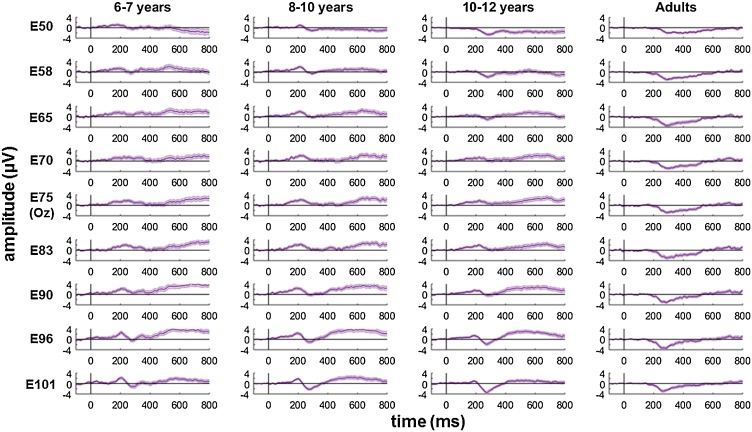
**Coherent motion evoked potentials in occipital electrodes.** Evoked potentials averaged for the highest coherence level (75%) in electrode 75 (E75; corresponding to Oz) and 4 electrodes laterally positioned to the right (E83, E90, E96, E101) and left (E70, E65, E58, E50), where E50 and E101 are the most laterally located electrodes. Shaded error bars represent the standard error of the mean.

Component 2 appears to resemble the previously reported coherence-onset visual evoked potential in adults, in three ways. First, in adults, the onset of coherent motion reportedly gives rise to a negative, N2 peak at about 300 ms in occipital electrodes, in the absence of a preceding positive, P1 peak (Niedeggen and Wist, 1999), similar to the adult waveform for component 2 (Fig. 3). Second, the amplitude of the N2 scales with motion coherence, with low coherence levels failing to elicit an observable visual evoked potential (Niedeggen and Wist, 1999; Patzwahl and Zanker, 2000), as we see in our component 2 waveform. Third, the coherence-onset visual evoked potential exhibits a predominance over right occipital electrodes (Niedeggen and Wist, 1999), as shown also in the topographic map for component 2. To compare component 2 with the coherence-onset visual evoked potential, we plotted the activity in electrode Oz and laterally positioned electrodes on either side, in the highest coherence (75%) condition (Fig. 4). This figure confirms that the adults show an N2 peaking at ˜300 ms in these electrodes, but no P1, in line with Niedeggen and Wist (1999). As children get older, there is a shift in dominance where the N2 becomes more pronounced while the amplitude of P1 reduces. Further topographical information, convergent with our reliable component analysis, is presented in Supplementary Fig. S2.

**Fig. 5:**
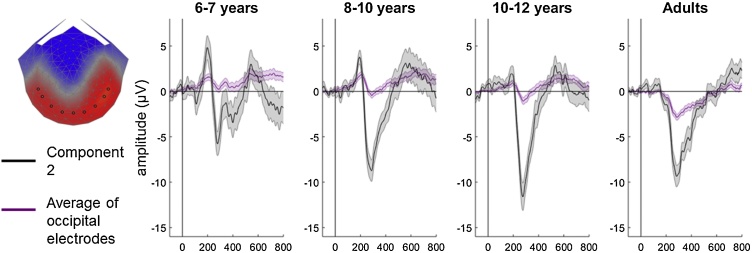
**Time-course of reliable component 2 and average across occipital electrodes.** The topography of component 2 and the selected occipital electrodes (black circles; from left-to-right: E50, E58, E65, E70, E75 (Oz), E83, E90, E96, E101) are shown in the left-most panel. In the remaining panels, the black line shows the averaged waveform for component 2 for each group in the highest coherence condition (75%). The purple line represents the waveform averaged over the selected occipital electrodes. Shaded error bars represent the standard error of the mean.

In Fig. 5, we have overlaid the waveform of component 2 and the average of the waveforms of the 9 occipital electrodes shown in Fig. 4. There are similarities in the overall time-course, with a high correlation between the group average waveforms (*r* = .68, *p* <  .001) although the amplitude of component 2 is larger. This amplitude difference may be attributable to the fact that the reliable components waveform is a weighted average of all electrodes, in which sensors other than the occipital electrodes are weighted highly in order to maximise trial-by-trial reliability, and because this averaging process increases signal-to-noise ratio.

### 3.4 Identifying decision-specific EEG activity

**Fig. 6:**
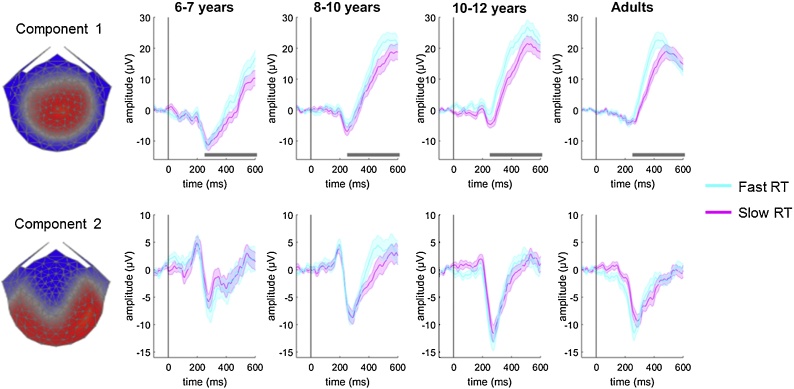
**Comparison of component waveforms for trials with fast and slow response times in the 75% coherence condition.** The left panel shows scalp topographies for the components. Each participant’s trials were median-split into those with fast and slow response times (RT) in the highest coherence (75%) condition. The rightmost panels show the component waveforms for fast (cyan) and slow (magenta) trials for each age group, with shaded bars representing the standard error of the mean. In component 1, a significant cluster for the effect of RT bin was found between 262 ms and 600 ms, as marked with grey horizontal bars.

So far, we have shown two EEG components that change with age, and we have shown concomitant age-related changes in behaviour. Inspection of the components suggests that component 1 has the hallmarks of a decision variable (as did component 1 extracted from data generated in a fine motion discrimination task by Dmochowski and Norcia, 2015). It scales with motion coherence and increases as a function of time, which suggests that it reflects accumulated evidence rather than just momentary evidence (Kelly and O’Connell, 2013). However, another key criteria of a decision-variable is that it should predict response time even when the strength of sensory evidence (i.e., coherence) is held constant (Kelly and O’Connell, 2013). To test this criterion, we sorted each participant’s correct and incorrect trials from the highest coherence level (75%) into fast and slow response time bins, using a median split, and compared the component waveforms for slow and fast responses (Fig. 6). We used 4 (group) x 2 (response time bin) cluster-corrected bootstrapped ANOVAs at each timepoint to investigate if and when the component waveforms were differentiated by response time. These analyses were conducted only on the time window between 100 ms preceding the stimulus onset and 600 ms following the stimulus onset as there were missing data beyond this window in the fast response time bin.

Component 1 showed a significant cluster for the effect of response time bin between 262 ms and 600 ms (shown on Fig. 6), and as expected, a significant cluster for the effect of group (between 200 ms and 600 ms), but no interaction between group and response time. The fast response trials had a more steeply rising component that reached higher amplitudes and peaked earlier than in the slow response trials. Component 2 showed no significant clusters for the effect of response time bin, nor for the interaction effect between response time bin and group. As expected, though, there were effects of group (between 162 ms and 290 ms). These results suggest that component 1 (but not component 2) reflects decision-specific activity.

## 4 Discussion

In this study, we investigated age-related changes in evoked responses to coherent motion onset in children aged 6–12 years and adults. As expected, clear behavioural improvements in coherent motion discrimination were apparent. The two youngest groups of children (6- to 7-year-olds and 8- to 10-year-olds) were significantly less accurate than adults, and all child groups including the 10- to 12-year-olds made significantly slower responses than adults. We identified two stimulus-locked reliable components that differed across age-groups: an early component over occipital electrodes (explaining the second-largest portion of the reliability) and a later, sustained component over centro-parietal electrodes (explaining the largest portion of the reliability).

We will begin by discussing the component that explained the second-largest portion of the reliability, which resembled previously reported coherent-motion onset visual evoked potentials in adults, in both its occipital topography and time-course. This component scaled with coherence, but did not predict response times independently of coherence, suggesting that it reflects early sensory encoding preceding the decision-making process. In adults, this component had a negative peak at ˜300 ms, like the coherence-onset N2 (Niedeggen and Wist, 1999; Patzwahl and Zanker, 2000). The amplitude of this negative peak was reduced in young children, which could reflect immaturities in extrastriate temporo-occipital areas, such as V5/MT (Aspell et al., 2005; Probst et al., 1993). In addition to this negative peak, the children also showed an initial positive peak at around ˜200 ms, which was not present in the adults. The relative dominance of the positive to negative peak shifted with age, and was still not fully adult-like in the oldest children. This child-specific positive peak seems similar to the P1 peak reported in response to motion-onset (Kuba et al., 2007), and indeed, the dominance of the motion-onset P1 has also been shown to reduce through childhood (Langrová et al., 2006). However, the P1 peak has been speculated to be pattern-specific, rather than motion-specific, so we did not expect it to appear in our paradigm designed to isolate motion-specific activity. Interestingly, this suggests that children are processing directional, coherent motion differently to adults as early as 160 ms. The lack of a response time effect for this component suggests that this early child-specific processing is related to the encoding of the stimulus, rather than the decision-making process. While children may have an additional source of neural activity that is inactive in adults, it is also possible that the P1 peak in adults is masked due to phase cancellation from the large negative peak, or because the positive and negative peaks overlap temporally in adults. Further measurements with EEG source imaging and/or functional MRI or MEG would help clarify how the underlying neural sources vary with age (Scerif et al., 2006).

The component that explained the most reliability was maximal over centro-parietal electrodes and exhibited a sustained, rising positivity. A rising positivity has been previously reported with coherent-onset visual evoked potentials in adults, and has been hypothesised to reflect the decision-making process (Niedeggen and Wist, 1999). Our component bore a striking resemblance to component 1 extracted by RCA in a previous EEG experiment in which adults performed a fine motion discrimination task (Dmochowski and Norcia, 2015). The authors of the previous study likened their component to a more traditional event-related potential measure, the centroparietal positivity (CPP; O’Connell et al., 2012; Kelly and O’Connell, 2013) – which in turn appears to resemble the P300/P3 (Nieuwenhuis et al., 2005; O’Connell et al., 2012; Twomey et al., 2015). Like the CPP, our component met Kelly and O’Connell’s (2013) three criteria for a decision variable: its activity a) ramped up over time, reflecting accumulated evidence rather than merely momentary evidence, b) scaled with the strength of sensory evidence (motion coherence), and c) predicted the timing of responses independently of the strength of sensory evidence. The amplitude of this sustained rising component increased with age in the child participants, but reduced slightly by adulthood, potentially reflecting a trade-off between passive differences in skull conductivity (see below) and neural differences with age. The component also rose more steeply with age, with the youngest children having shallower slopes than the older children and adults. These age-related changes may reflect developmental changes in the rate that parietal areas can accumulate evidence towards a decision bound (Shadlen and Newsome, 2001) – a possibility that we will investigate in future modelling studies.

It is important to consider whether the age-related changes we observe in these reliable components could arise merely as a result of developmental changes in skull conductivity with age (Hoekema et al., 2001; Wendel et al., 2010; see also Scerif et al., 2006, for review). Increased skull conductivity in young children would lead to increased amplitudes, but our centro-parietal component showed the opposite effect, with larger amplitudes in older children. However, we note that amplitudes in 10- to 12-year-old children are slightly reduced compared to adults, which could be in part due to changes in skull conductivity. In the occipital component, the shape of the component waveform varied between age-groups, and while the amplitude of the initial, positive peak reduced with age, the amplitude of the negative peak *increased* with age. We therefore conclude that age-related differences in these components cannot be explained by developmental changes in skull conductivity, and instead reflect developmental changes in neural activity.

In this study, we used a dimension-reduction technique, RCA, which has not been used previously to investigate development over childhood. RCA identifies activity that is reliably evoked across trials, which has some similarities to computing average waveforms over a subset of electrodes, but offers three potential benefits over the more traditional analysis approach. First, components are identified in a data-driven way, rather than the researcher selecting electrodes to focus on, either post-hoc or based on previous literature. Second, RCA makes use of all electrodes, thus maximising the signal-to-noise ratio – a particular advantage when analysing high-density recordings. Third, RCA can identify multiple biologically plausible components that likely correspond to distinct neural sources. To compare the waveforms produced by RCA, we used a mass univariate statistical analysis to investigate effects at all timepoints along the component waveforms, which avoided the need to focus only on mean or peak amplitudes within defined windows (Groppe et al., 2011; Pernet et al., 2011). Our study confirms that RCA can be used to study development through childhood and produces components that are meaningful in relation to previously identified event-related potential components. Note that here we identified components with spatial weights in adults and used these for projecting through data from the other groups. This approach resembles identifying regions of interest, which may explain why it relates well to previous event-related potential research, but differs from investigating changes in topography over time within a group of participants (e.g., Brunet et al., 2011; Murray et al., 2008), which may offer complementary insights.

Our results show that developments in coherent motion performance through childhood are accompanied by changes in neural activity, and that age-related changes continue at least until 10–12 years of age. In a previous behavioural study, we reported that age-related changes in motion coherence sensitivity were driven by improvements in the ability to average motion information, rather than age-related reductions in local, internal noise (Manning et al., 2014). The current study isolates and reveals age-related differences in coherence-specific responses, which is also in line with an interpretation that motion integration abilities continue to develop throughout childhood. Interestingly, our results show age-related changes in both early occipital and later centroparietal components, which we speculate reflects changes in both sensory encoding and the accumulation of information towards a decision bound. A recent study by Braddick et al. (2016) supports this interpretation by suggesting that the decision-making process may be important for explaining individual differences in children’s coherent motion thresholds, with lower thresholds being associated with a larger parietal lobe surface area, rather than differences in extrastriate areas. Together, our results suggest that young children may be limited by their ability to accumulate information towards a decision bound.

To conclude, our results show clear age-related changes in two reliable components locked to the onset of coherent motion in children aged 6–12 years and adults. Elevated motion coherence thresholds have been reported in a range of neurodevelopmental conditions, such as autism (Manning et al., 2013; Pellicano et al., 2005), dyslexia (Demb et al., 1998; Hansen et al., 2001), Williams Syndrome (Atkinson et al., 1997, 2006) and Fragile X Syndrome (Kogan et al., 2004). The protracted development in neural correlates reported here could leave motion processing vulnerable to atypical development (see also Braddick et al., 2003). Interestingly, the fact that our EEG components show distinct developmental changes leaves open the possibility that elevated motion coherence thresholds could arise for different reasons in different developmental conditions. Future studies investigating coherence onset visual evoked potentials in different conditions will help to address this question.

## Appendix A Supplementary data

Supplementary Video S1 Stimulus video: 10% coherence.

Supplementary Video S2 Stimulus video: 30% coherence.

Supplementary Video S3 Stimulus video: 50% coherence.

Supplementary Video S4 Stimulus video: 75% coherence.

## Footnotes

2 Degrees of freedom are reported with a Greenhouse-Geisser correction when Mauchly’s test indicated a violation of the assumption of sphericity (p < .05).
