# Corrigendum to “Neural dynamics underlying coherent motion perception in children and adults” [Dev. Cogn. Neurosci. 38 (August) (2019) 100670]

**DOI:** 10.1016/j.dcn.2019.100748

**Published:** 2019-12-23

**Authors:** Catherine Manning, Blair Kaneshiro, Peter J. Kohler, Mihaela Duta, Gaia Scerif, Anthony M. Norcia

**Affiliations:** a Department of Experimental Psychology, University of Oxford, Anna Watts Building, Radcliffe Observatory Quarter, Woodstock Road, Oxford, OX2 6GG, UK; b Department of Otolaryngology Head and Neck Surgery, Stanford University School of Medicine, Stanford University, 2452 Watson Court, Palo Alto, CA, 94303, USA; c Department of Psychology, Stanford University, Jordan Hall, 450 Serra Mall, Stanford, CA, 94305, USA

The authors regret that there was an error in the analysis code for this paper, where we had put the X and Y variables in the wrong order using the MATLAB regression function. The corrected code has been uploaded onto the OSF website: https://osf.io/fkjt6/. This error affects only a small part of the analysis, and all of our conclusions stand. The exact values of the corresponding statistical tests have changed, however, with the effect sizes being much bigger with the corrected analysis code. We still find evidence for significant effects of coherence and group, with steeper slopes in easier conditions than hard conditions, and steeper slopes in adults than young children.

The corrected values are presented in the following paragraph:

Inspection of Figure 3 also suggested that the slope of increasing positivity became steeper as a function of age. To quantify this, we fit a linear regression to each observer’s average component waveform between 260 ms and 460 ms after stimulus onset in each coherence condition and conducted a mixed group by coherence condition ANOVA on the slope coefficients. This revealed a main effect of coherence, F(2.08, 245.17) = 213.18, p < .001, η_p_^2^ = .64, with shallower slopes in the 10 % coherence condition (M = .01, SE = .005) than in the higher coherence conditions (30 %: M = .07, SE = .007, p < .001; 50 %: M = .16, SE = .009, p < .001; 75 %: M = .22, SE = .01, p < .001). There was also a significant main effect of group, F(3,118) = 9.01, p < .001, η_p_^2^ = .19, with the youngest, 6- to 7-year-old children having shallower slopes (M = .06, SE = .01) than the adults (M = .15, SE = .016), p < .001, while the slopes of the older children did not differ significantly from those of adults (8- to 10-year-olds: M = .11, SE = .012, p = .10; 10- to 12-year-olds: M = .13, SE = .012, p = .38). The interaction between group and coherence level was also significant, F(6.23, 245.17) = 5.55, p < .001, η_p_^2^ = .12, with clearer group differences in the higher coherence conditions than in the lowest (10 %) coherence condition.

The authors would like to apologise for any inconvenience caused.

