# Supplementary material for: Neural dynamics underlying coherent motion perception in children and adults
Source: Dev Cogn Neurosci. 2019 Jun 13;38:100670. doi: 10.1016/j.dcn.2019.100670 (PMC6688051; doi:10.1016/j.dcn.2019.100670)
Supplement: Supplementary file 1 [file mmc1.docx]

**Supplementary Material**


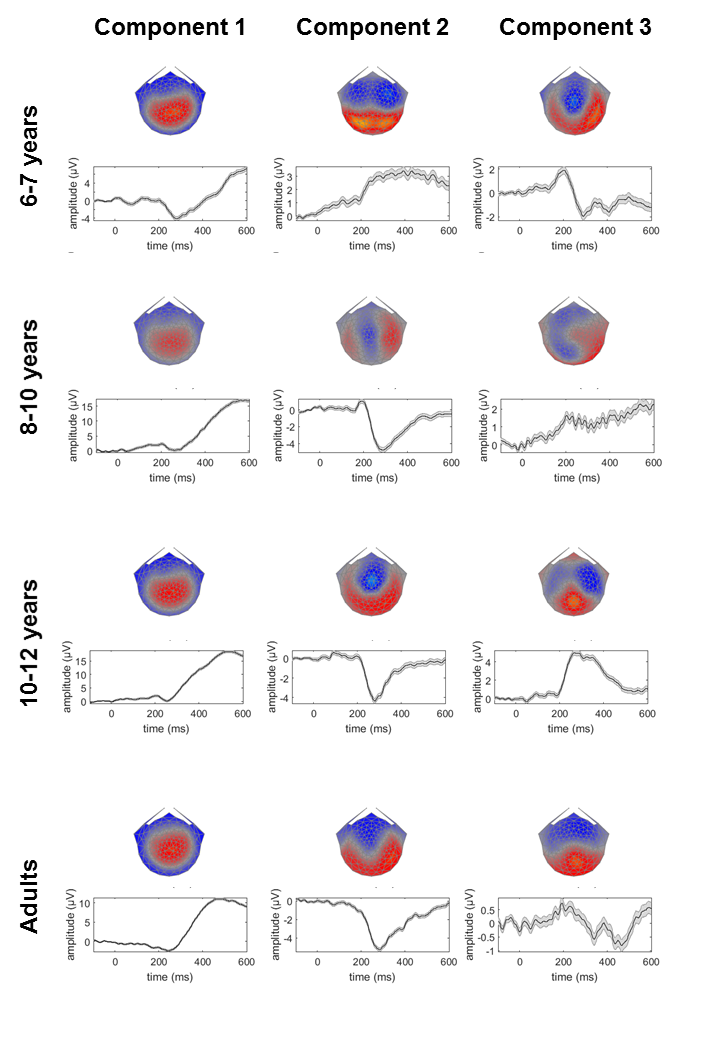


**Figure S1. Scalp topographies and waveforms for components 1-3 for each group.** Topographic visualisations of the forward-model projections of components 1-3 following reliable components analysis (RCA) on each age group’s data, pooled across coherence conditions. The waveforms show the data multiplied by these spatial weights. Shaded error bars represent the standard error of the mean.

**
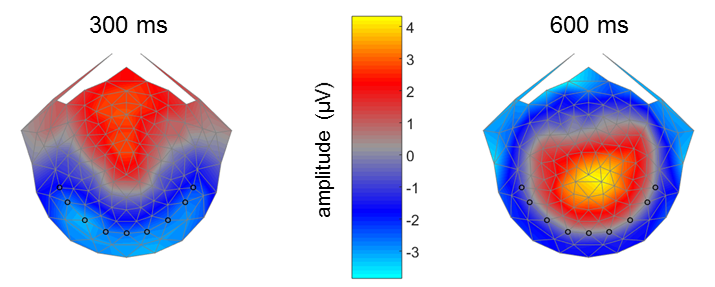
**

**Figure S2. Topographic maps of electrode amplitudes at 300 ms and 600 ms after coherence onset**

Amplitudes for each electrode averaged across adult participants for the highest coherence level (75%) at 300 ms (left panel) after coherence onset, which is when the coherence-onset visual evoked potential has been reported previously to peak (Niedeggen & Wist, 1999), and at 600 ms (right panel) after coherence onset, as the coherence-onset visual evoked potential becomes increasingly positive. Black circles highlight the selected occipital electrodes for the coherent motion evoked potential analysis (from left-to-right: E50, E58, E65, E70, E75 (Oz), E83, E90, E96, E101). As expected, at 300 ms there are negative amplitudes over occipital electrodes (including those selected for the coherence-onset visual evoked potential analysis). By 600 ms, the central electrodes show more positive amplitudes, in line with the conclusions from our reliable component analysis.
